# A Study on the UV Degradation Performance of Rhodamine B by Zn-TiO_2_ Photocatalysts and Cement Mortar-Based Zn-TiO_2_ Composites

**DOI:** 10.3390/ma19061094

**Published:** 2026-03-12

**Authors:** Peng Wang, Zihao Jiang, Lanshuo Xing, Jiale Xiao, Ze Wu, Haiyang Chen, Yichen Xu, Hai Wang

**Affiliations:** 1 School of Civil and Engineering, Hebei University of Architecture, Zhangjiakou 075000, China; jzh05130221@163.com (Z.J.); xlshuo0121@163.com (L.X.); 15152035811@163.com (J.X.); 15633097385@163.com (Z.W.); chenhaiyang_123@163.com (H.C.); 2 Hebei Key Laboratory of Diagnosis, Reconstruction and Anti-Disaster of Civil Engineering, Zhangjiakou 075000, China; 3 Institute of Structured and Architected Materials, Liaoning Academy of Materials, Shenyang 110167, China; ycxu@lam.ln.cn; 4 Shenyang National Laboratory for Materials Science, Institute of Metal Research, Chinese Academy of Sciences, Shenyang 110016, China

**Keywords:** orthogonal experimental design, hydrothermal method, Zn-TiO_2_ catalyst, photo-functional building materials, air-entrained composite mortar matrix

## Abstract

Zn-TiO_2_ composites were synthesized via a hydrothermal method, and their photocatalytic performance was optimized using an orthogonal design. Among the factors of hydrothermal temperature, reaction time, and Ti/Zn molar ratio, hydrothermal temperature showed the most significant influence on the photocatalytic performance of Zn-TiO_2_. The Zn-TiO_2_ obtained under the optimal conditions (120 °C, 10 h, and a Ti/Zn molar ratio of 100:5) exhibited the best photocatalytic performance, with a 26% improvement in the photocatalytic degradation efficiency of Rhodamine B (RB) compared to pure TiO_2_ under identical conditions. The composition, morphology, and structure of the Zn-TiO_2_ photocatalysts were characterized by XRD, SEM-EDS, N_2_ adsorption–desorption, and XPS, thereby enabling analysis of the mechanism for the enhancement of its photocatalytic performance. In this work, air-entrained composite mortar (ACM) with a double-layer structure was designed as a contrast to conventional cement mortar (CM). Novel green building materials with pollutant-degradation capability were developed by loading Zn-TiO_2_ and TiO_2_ photocatalysts onto these different mortar surfaces. Photocatalytic tests and cyclic aging experiments demonstrated that the Zn-TiO_2_/ACM achieved the superior degradation effect on the RB solution and maintained good catalytic stability. These findings suggest broad application prospects in the field of green building materials.

## 1. Introduction

In recent years, with the acceleration of industrialization and urbanization, distinct deterioration in air and water quality has emerged globally. In particular, high emissions of gaseous pollutants, such as nitrogen oxides and volatile organic compounds, pose serious threats to human health and ecological safety [1]. Traditional pollution control methods, including chemical oxidation, adsorption, physical filtration, and biochemical treatments, often suffer from limitations such as high costs, high energy consumption, difficulty in efficiently removing refractory organics and trace pollutants, and the potential generation of secondary pollutants [2]. Consequently, there is an urgent need in research and engineering to explore green treatment techniques featuring low energy costs, reduced secondary impacts, and sustained effectiveness under passive or on-site conditions. Photocatalytic technology is widely regarded as a promising and sustainable approach for environmental purification. This is attributed to its ability to drive the redox reactions for the decomposition of pollutants using light energy at ambient temperature, generating controllable reactive oxygen species without typically producing persistent harmful by-products [3,4].

With the growing demand for green building materials and urban air quality improvement, incorporating photocatalysts into or coating them onto cement-based materials to achieve functions such as pollutant degradation, self-cleaning, and antibacterial effects has become a significant development trend in the construction field [5]. Currently, the primary implementation methods for photocatalytic building materials include: direct mixing of photocatalytic nanoparticles into concrete or mortar, surface coating or loading on structural components, and the construction of photoactive coatings via sol–gel or surface modification techniques [6,7]. These methods have demonstrated certain environmental purification capabilities (e.g., degradation of NOx and organic matter) in applications such as exterior walls, paving blocks, and prefabricated components [8]. However, several challenges remain in practical engineering applications. For example, photocatalytic particles are prone to agglomeration in alkaline cement pastes or become encapsulated by hydration products, leading to insufficient exposure of active species [9]. Moreover, the catalytic activity significantly attenuates under the combined effects of prolonged light exposure, rainfall erosion, and pollutant coverage. Furthermore, the interfacial bonding state, dispersion uniformity, and the impacts on rheological and mechanical properties have also limited the large-scale engineering application of these materials [10]. Therefore, the development of materials that combine high photocatalytic activity, excellent dispersion, and compatibility with cement systems is crucial for advancing the engineering application of photo-functional cementitious materials.

Titanium dioxide (TiO_2_) was widely used as a photocatalytic material due to its advantages such as structural stability, low cost, and environmental friendliness [11]. However, pure TiO_2_ was still confronted by two critical limiting factors. First, its bandgap energy is approximately 3.2 eV, which primarily relies on ultraviolet (UV) light excitation; moreover, its rapid charge recombination rate leads to relatively low quantum efficiency [12]. Second, although defect engineering (e.g., oxygen vacancies and Ti^3+^) can enhance electron migration and surface activity, its structural stability remains uncertain under long-term environmental exposure [13]. In practical engineering applications, such as the photocatalytic self-cleaning or pollutant degradation functions of concrete, mortar, and pavement materials, the efficiency of TiO_2_ was often constrained by illumination conditions, interfacial adsorption behavior, particle dispersion, and the surface state of the material [7]. Consequently, relying on unmodified TiO_2_ was insufficient to sustain continuous and high-efficiency UV photocatalytic performance within cementitious systems. This drove the development of modification strategies such as heterostructure construction, metal-ion doping, and interfacial engineering regulation [14].

Studies showed that the heterostructures formed by TiO_2_ and ZnO could create a spatial charge separation barrier at the interface, effectively inhibiting the recombination of photogenerated electron–hole pairs and thereby significantly enhancing photocatalytic efficiency under UV illumination [15]. Further research indicated that loading ZnO nanoparticles onto the TiO_2_ surface could facilitate multi-level interfacial electron transfer, thereby improving both photocatalytic activity and stability [16]. Additionally, Zn-doping of TiO_2_ with the precise exposure of specific crystal facets (e.g., the (001) facet) was proven to regulate surface energy bands and further suppress carrier recombination [17]. In cementitious material applications, the Zn-TiO_2_ system demonstrated favorable photocatalytic activity and engineering stability. For instance, when Zn-Ti composite oxides were loaded onto the surface of cement clinker, the photocatalytic performance of the material could be significantly improved without markedly affecting the hydration behavior [10]. When incorporated into high-performance concrete, ZnO and Zn-TiO_2_ nanoparticles could simultaneously enhance the pollutant degradation capacity of the material, providing an experimental basis for the engineering application of photo-functional concrete [18]. Meanwhile, Zn-TiO_2_ mixed powders provided additional active sites within the cement paste and enhanced photocatalytic activity without significantly compromising rheological properties [9]. Zn-doping or Zn-TiO_2_ compositing held substantial research value for photocatalytic cementitious materials, offering advantages in both cost and performance [6].

Adding photocatalysts to mortar to prepare self-cleaning mortar is a research hotspot in the field of building materials. For instance, partially substituting Portland cement with TiO_2_–silica fume composite powder, which has good pozzolanic activity, can result in mortar with a self-cleaning function and enhanced compressive strength [19]. Adding functionalized nanoparticles, such as TiO_2_ and ZnO, to the geopolymer cement prepared from fly ash can result in the production of a green self-cleaning coating [20]. However, the method of incorporation and the ratio directly affect the dispersion of nanoparticles in the cement matrix, thereby limiting the performance. For a cement mortar-based Zn-TiO_2_ material under UV irradiation, the actual photocatalytic efficiency and long-term stability still require further research. The intrinsic catalytic performance of Zn-TiO_2_, its binding mechanism with the cementitious matrix and the doping ratio all significantly influence the engineering service performance of photo-functional building materials. Herein, the preparation conditions of the Zn-TiO_2_ catalyst were optimized through orthogonal experiments, and the optimized Zn-TiO_2_ catalyst was applied as a coating on the surface of a double-layer structured air-entrained composite cement mortar, yielding a novel photocatalytic building material. The results indicated that the design of the substrate structure improved the dispersion of the catalyst and the interfacial adhesion, thereby enhancing the photocatalytic activity of the building material. Furthermore, the composite exhibited good cyclic stability. This substrate structural design and interfacial binding approach are also applicable to other construction materials such as concrete, which can facilitate the engineering application of photo-functional building materials.

## 2. Experimental Section

### 2.1. Hydrothermal Preparation of Zn-TiO_2_ Composites

The main raw materials involved in the preparation of Zn-TiO_2_ were as follows: zinc sulfate heptahydrate (ZnSO_4_·7H_2_O, analytically pure, Sinopharm Chemical Reagent Co., Ltd., Shanghai, China) and sodium hydroxide (NaOH, analytically pure, Sinopharm Chemical Reagent Co., Ltd., Shanghai, China). The TiO_2_ powder was purchased from Guangzhou Metal Metallurgy (Group) Co., Ltd (Guangzhou, China). The deionized water used in this work was homemade in the laboratory.

#### 2.1.1. Preparation of Precursor Solution

According to the experimental design, the predetermined amounts of ZnSO_4_ solution (0.1 mol/L) and TiO_2_ powder were added to a beaker and stirred for 15 min. Subsequently, a predetermined volume of NaOH solution (1 mol/L) was added dropwise to the suspension, followed by continued stirring for 60 min to ensure complete hydrolysis of Zn^2+^ and formation of Zn(OH)_2_. The precursor solution was then ultrasonically dispersed for 10 min to enhance uniformity.

#### 2.1.2. Hydrothermal Reaction Process

The solution was poured into the hydrothermal reactor and sealed. Then it was placed in a drying oven. The hydrothermal reaction was carried out at temperatures ranging from 100 °C to 180 °C, with the reaction time varying from 4 h to 12 h. The high temperature and pressure conditions facilitated precursor hydrolysis, nucleation and crystal growth, resulting in the formation of stable Zn-TiO_2_ composites. After completion of the reaction, the reactor was naturally cooled to room temperature. The precipitate was collected by centrifugation, washed sequentially with deionized water and anhydrous ethanol until neutral (pH ≈ 7), and dried in an oven at 60 °C for 12 h to yield Zn-TiO_2_ powder.

### 2.2. Orthogonal Experimental Design

During the preparation of Zn-TiO_2_ photocatalysts, factors such as hydrothermal temperature, reaction time, and Ti/Zn molar ratio significantly affect the crystal size, microstructure, and morphology of the resulting product. Therefore, an orthogonal experimental design was adopted to determine the optimal preparation conditions [21]. The hydrothermal reaction temperature, reaction time, and Ti/Zn molar ratio were selected as the factors, with the factor levels presented in Table 1. Based on the factor levels in Table 1, the preparation conditions for the Zn-TiO_2_ photocatalysts were determined. The detailed design of the orthogonal experiment is shown in Table 2.

### 2.3. Evaluation of Zn-TiO_2_ Photocatalytic Performance

RB (analytically pure, Sinopharm Chemical Reagent Co., Ltd., Shanghai, China) was employed as the model pollutant [22,23], with a 365 nm UV lamp (Shenzhen Xinyi Optoelectronics Ltd., Shenzhen, China) used to simulate ultraviolet irradiation. The distance from the light source to the solution was 30 mm and the power of the UV lamp was 3 W. The experimental procedure was as follows: First, 40 mL RB solution (20 mg/L) was added to a beaker, followed by the addition of 0.04 g Zn-TiO_2_ photocatalyst powder. Before irradiation, the beaker was fully wrapped with aluminum foil and placed on a magnetic stirrer for dark treatment for 15 min to reach adsorption–desorption equilibrium [24]. The absorbance *A*_1_ of the supernatant was measured using a UV–Vis spectrophotometer (Beijing Scan Research and Study Technology Ltd., Beijing, China), and then the solution was returned. Subsequently, the UV lamp was positioned vertically above the beaker, and the solution was irradiated under continuous stirring for 15 min to measure the absorbance *A*_2_ of the supernatant. The irradiation was continued for another 45 min and the above steps were repeated three times to determine the photocatalytic activity of Zn-TiO_2_ [25,26]. The photocatalytic degradation efficiency of the RB solution using different samples under UV irradiation can be calculated by Formula (1):

(1) $$D=\frac{A_{0}-A_{5}}{A_{0}}\times 100\%$$

where *D* represents the photocatalytic degradation efficiency, *A*_0_ is the initial absorbance of the RB solution, and *A*_5_ is the absorbance measured after the reaction stopped [7].

### 2.4. Preparation and Performance Testing of Cement Mortar-Based Zn-TiO_2_ Composites

The main raw materials involved in the preparation of cement mortar-based Zn-TiO_2_ composites were as follows: sodium dodecyl sulfate (C_12_H_25_NaO_4_S, chemical pure, Sinopharm Chemical Reagent Co., Ltd., Shanghai, China) and sodium metaphosphate (Na_6_O_18_P_6_, 65%, Shanghai Macklin Biochemical Technology Co., Ltd., Shanghai, China). The P·O 42.5 grade ordinary Portland cement was purchased from Xuanhua Jinyu Cement Co., Ltd. (Zhangjiakou, China). The sand was purchased from Xiamen ISO Standard Sand Co., Ltd (Xiamen, China).

#### 2.4.1. Types and Preparation of Cement Mortar Specimens

To systematically investigate the influence of matrix characteristics on photocatalyst loading efficiency and performance, two different types of cement mortar matrices were designed and prepared in this study: reference cement mortar (CM) and air-entrained composite mortar (ACM) with a modified surface layer structure.

CM specimens with a diameter of 4.2 cm and a height of 2.5 cm were prepared using the mix ratio of cement: sand: water = 1:4:0.6. The specific procedure was as follows: Sand and cement were added to the stirring pot and dry-mixed for 30 s to ensure uniform mixing of aggregates and cementitious materials. Water was then added according to the mix ratio while stirring for 2–3 min simultaneously. The mixture was placed in the mold in two steps to ensure proper compaction, then leveled off and transferred to the curing room for curing.

**Figure 1:**
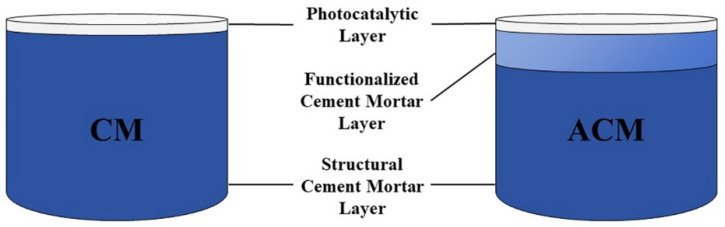
Schematic structure of two types of cement mortar-based photocatalytic composites.

To improve the surface microstructure and enhance photocatalyst loading capacity, a double-layer composite structure was designed, consisting of a base layer accounting for 4/5 of the total height (with the same mix ratio as the reference cement mortar) and a functionalized surface layer accounting for 1/5 (incorporating 0.06% air-entraining agent C_12_H_25_NaO_4_S according to the mass of cementitious material). The base layer was prepared using the same method as described above. The preparation procedure for the functionalized surface layer was as follows: A predetermined amount of air-entraining agent was first dissolved in the weighed water according to the mix ratio. Sand and cement were added to the mixing pot and dry-mixed for 30 s. The water containing the air-entraining agent was then slowly and evenly added while stirring synchronously for 2–3 min. Before the base layer reached initial setting, the freshly mixed slurry was poured into the remaining 1/5 of the mold volume, compacted on a vibrating table, leveled off, and transferred to the curing room for curing. Figure 1 presents a schematic diagram of the structures of the two types of cement mortar-based photocatalyst composites described above.

Meanwhile, CM and ACM blocks (160 mm × 40 mm × 40 mm) were prepared using the same methods and conditions and cured in the curing room for 28 days. The compressive strengths of CM and ACM were 20.6 MPa and 24.7 MPa respectively. The test result of ACM was 19.9% higher than that of CM, indicating that the air-entrained layer could enhance the mortar’s compressive strength, which is beneficial for the engineering application of ACM-based composites.

#### 2.4.2. Loading Method of Photocatalyst

A mass of 0.25 g dispersant (Na_6_O_18_P_6_; 10% of the sample mass) was dissolved in 25 mL deionized water. Under continuous stirring, 2.5 g of Zn-TiO_2_ powder was slowly added in batches to the dispersant solution. Subsequently, the solution was treated in an ultrasonic cleaner for 20–40 min, during which the temperature was maintained below 40 °C to prevent particle re-agglomeration or dispersant failure caused by excessive heat. After sonication, 2 mL slurry was uniformly coated onto the surfaces of the prepared CM and ACM, followed by drying at 60 °C for 2 h. The control group loaded with pure TiO_2_ was prepared using the same method.

#### 2.4.3. Photocatalytic Performance Testing of Composite Materials

**Figure 2:**
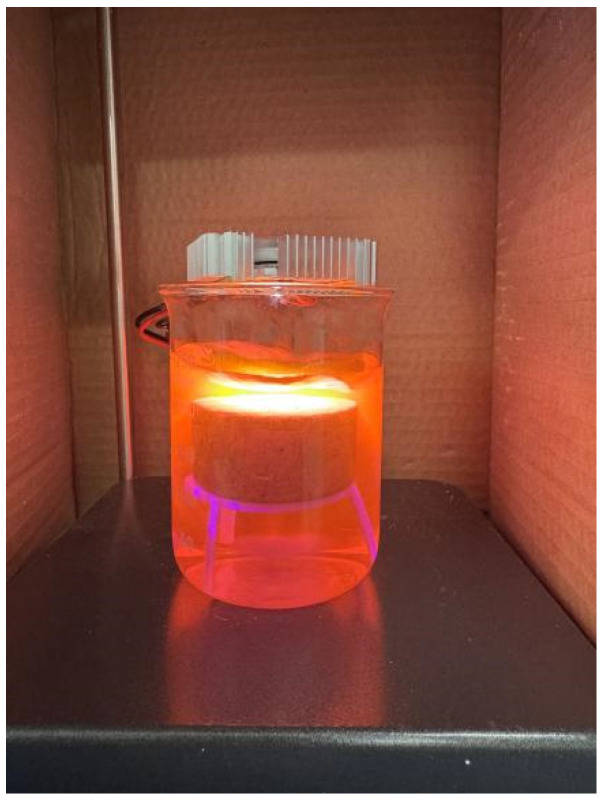
Photocatalytic performance testing setup of cement mortar-based catalysts.

RB solution was used as the model pollutant, and a 365 nm UV lamp was utilized to simulate ultraviolet radiation. A total of 40 mL RB solution (20 mg/L) was poured into a beaker, into which the cement mortar specimen and a hollow base (to provide space for the magnetic stirrer) were placed. The procedures for dark treatment and absorbance measurement were identical to those described above. The entire experimental setup was placed in a dark box, as illustrated in Figure 2.

## 3. Results and Discussion

### 3.1. Results and Range Analysis of Orthogonal Experiments of Zn-TiO_2_ Catalysts

**Figure 3:**
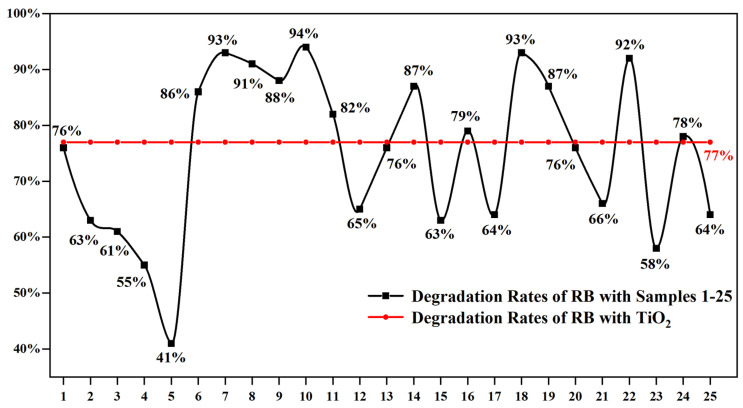
Degradation rates of RB solution with samples 1#–25# and TiO_2_ after 60 min of light irradiation.

The photocatalytic activities of samples 1#–25# obtained from the orthogonal experiments were evaluated using the degradation rate of RB solution after 60 min of UV irradiation, and the results are shown in Figure 3. It can be observed that the degradation rate of RB with TiO_2_ after 60 min of irradiation was 77%. In contrast, the degradation rates of the Zn-TiO_2_ composite materials ranged from 41% to 94%, with 48% of the samples exhibiting superior activity compared to TiO_2_. This indicates that the processing parameters significantly influence the activity of photocatalysts.

To determine the optimized preparation process for Zn-TiO_2_ composites, a range analysis was systematically conducted based on the orthogonal experimental results in Figure 3 to evaluate the degree of influence of each factor [21]. According to the range analysis results listed in Table 3, the order of influence of each factor on the photocatalytic activity is hydrothermal temperature > Ti/Zn molar ratio > reaction time. Based on the level response results of these three factors, the optimal combination was identified as: T = 120 °C, t = 10 h, and Ti/Zn molar ratio = 100:5.

### 3.2. Characterization of Zn-TiO_2_ Catalysts

**Figure 4:**
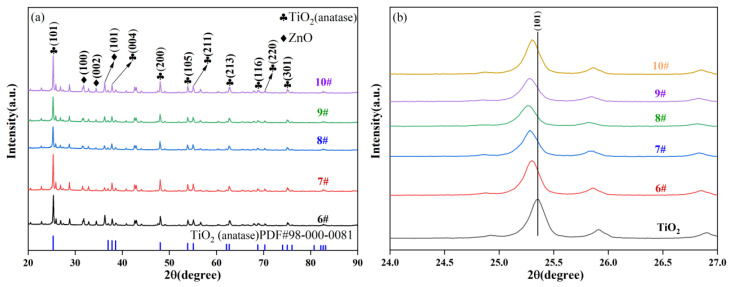
(**a**) Full XRD patterns and (**b**) magnified XRD patterns of Zn-TiO_2_ catalysts prepared at a hydrothermal temperature of 120 °C.

Five samples prepared with different Ti/Zn ratios at a hydrothermal temperature of 120 °C were characterized by XRD, and the results are shown in Figure 4. As observed in Figure 4a, the XRD patterns of 6#–10# all exhibit diffraction peaks corresponding to the (101), (004), (200) and other crystal planes of TiO_2_ (anatase). Anatase is the crystalline phase of TiO_2_ known for its high photocatalytic activity, indicating that TiO_2_ retains its anatase structure after Zn-doping modification, thus preserving its intrinsic catalytic foundation. Furthermore, diffraction peaks corresponding to the (001), (002), and (101) crystal planes of ZnO are also present in the XRD patterns, indicating that Zn exists in the form of ZnO within the composite, thus successfully forming a TiO_2_-ZnO composite structure. However, when the Ti/Zn molar ratio is excessively low (i.e., Zn content is too high), strong diffraction peaks associated with Zn impurities appear in the XRD patterns. The formation of large ZnO agglomerates may disrupt the effective crystal structure of the original Zn-TiO_2_ composite, leading to a reduction in photocatalytic performance. Simultaneously, the diffraction peaks of TiO_2_ exhibit a slight leftward shift or broadening, as shown in Figure 4b. This phenomenon is attributed to the fact that the ionic radius of Zn^2+^ is larger than that of Ti^4+^. Zn^2+^ enters the TiO_2_ lattice through interstitial or substitutional doping, replacing Ti^4+^ sites or occupying lattice interstices, thereby inducing lattice expansion and an increase in d-spacing within the TiO_2_ crystals. This not only provides more active sites for the photocatalytic reaction but also helps inhibit the recombination of photogenerated electrons and holes [27].

**Figure 5:**
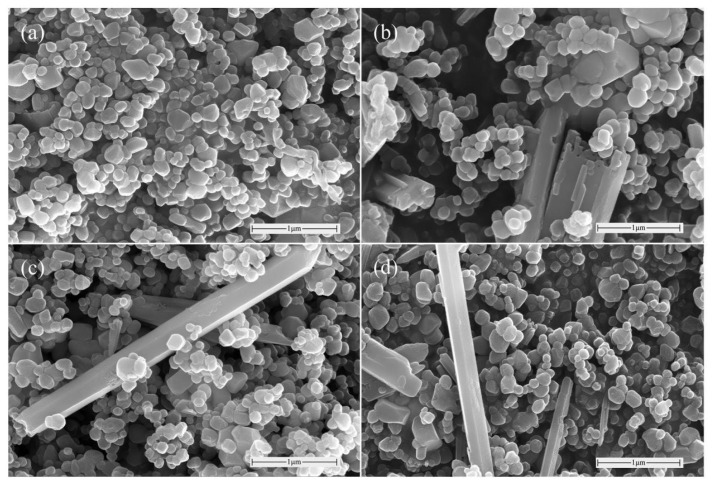
SEM images of different catalyst samples: (**a**) pure TiO_2_, (**b**) 5#, (**c**) 10#, and (**d**) 20#.

SEM was used to investigate the influence of hydrothermal process parameters on the microstructure and morphology of Zn-TiO_2_ catalysts prepared by the orthogonal experiments. Orthogonal analysis indicated that reaction time had the minimal impact on photocatalytic activity. Therefore, samples 5#, 10#, and 20#—prepared with a fixed reaction time of 12 h but varying hydrothermal temperatures and Ti/Zn molar ratios—were selected for comparative analysis with pure TiO_2_. The SEM images are shown in Figure 5.

In Figure 5a, the pure TiO_2_ consists of dense spherical nanoparticles with smooth surfaces and minor local agglomeration. After introducing ZnO, the TiO_2_ particle morphology changed markedly. At 100 °C and Ti:Zn = 100:25, ZnO aggregated in long rod-like forms and bound to TiO_2_ particles [28]. The lower hydrothermal temperature restricted grain migration and diffusion, while the excessively low Ti/Zn ratio caused incomplete ZnO growth along specific crystal directions and severe agglomeration, as shown in Figure 5b. This resulted in a substantial loss of active sites and severely reduced photocatalytic performance. When the temperature was raised to 120 °C with Ti:Zn = 100:5, ZnO retained its long rod-like morphology. The appropriate temperature improved the dispersibility of the crystals, preventing excessive agglomeration of ZnO. Some TiO_2_ nanoparticles adhered to the rod surfaces, as shown in Figure 5c, providing more active sites for photocatalytic reactions. When the temperature was further increased to 160 °C with Ti:Zn = 100:15, the high temperature accelerated particle agglomeration and enlarged particle size. The long rod-like ZnO and TiO_2_ were cross-stacked, as shown in Figure 5d. This covering effect likely reduced the light-absorption active sites of TiO_2_, further decreasing the photocatalytic performance of the sample [12].

### 3.3. In-Depth Characterization of the Structure and Composition of the Optimal Catalyst

The optimal Zn-TiO_2_ catalyst, recorded as sample 26#, was synthesized using the optimized processing parameters: a hydrothermal temperature of 120 °C, a reaction time of 10 h, and a Ti/Zn molar ratio of 100:5.

**Figure 6:**
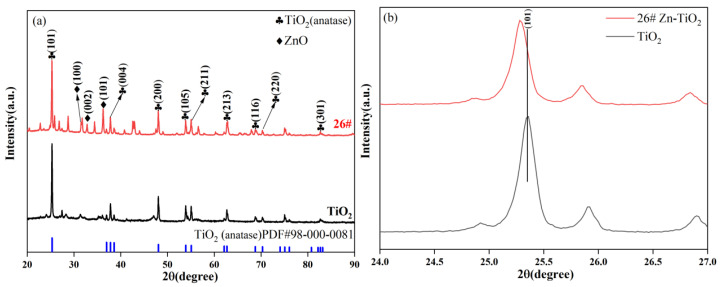
XRD patterns of pure TiO_2_ and sample 26#: (**a**) full patterns and (**b**) magnified patterns.

Figure 6a presents the XRD patterns of sample 26# and pure TiO_2_. Both patterns exhibit characteristic diffraction peaks of anatase TiO_2_. This indicates that TiO_2_ maintains an anatase crystalline structure after Zn-doping modification, which provides the foundation for high photocatalytic activity. Furthermore, characteristic diffraction peaks of ZnO are also observed in the XRD pattern of sample 26#. Simultaneously, Figure 6b shows that the TiO_2_ characteristic peaks of sample 26# slightly shifted to the left, suggesting that Zn^2+^ partially replaces the position of Ti^4+^ and exists in the form of ZnO. This confirms the successful synthesis of a TiO_2_-ZnO heterostructure [29]. This structure can not only refine the grain size of TiO_2_ and increase the exposure of active catalytic sites but also inhibit the recombination of photogenerated electron–hole pairs, thereby further enhancing photocatalytic efficiency.

**Figure 7:**
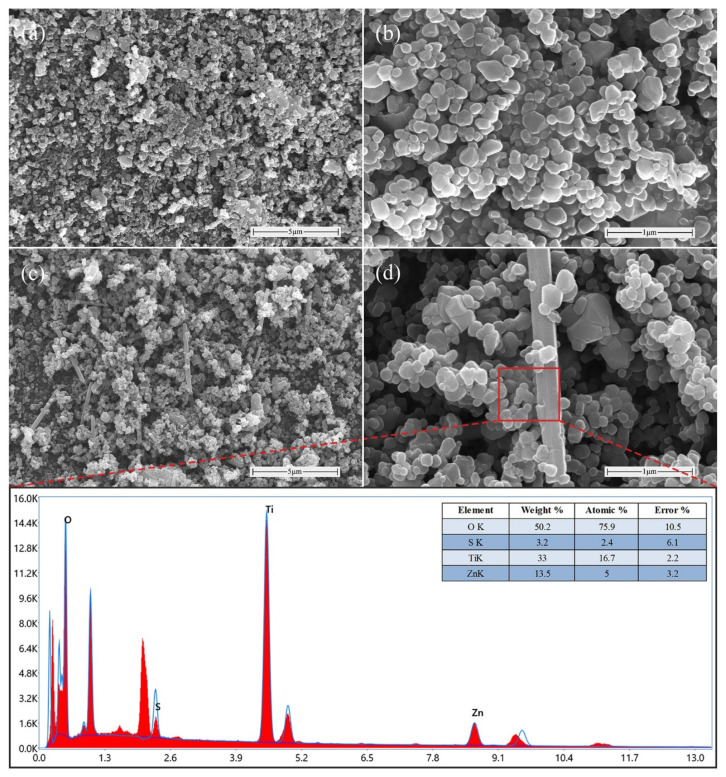
SEM-EDS characterization of the catalysts: (**a**,**b**) TiO_2_; (**c**,**d**) sample 26#.

The SEM images of TiO_2_ and sample 26# are shown in Figure 7. From Figure 7a,b, pure TiO_2_ appears as dense spherical nanoparticles with smooth surfaces and minor local agglomeration; the particles are mutually stacked, resulting in a relatively compact overall structure with limited active sites, which is unfavorable for photocatalytic reactions. In contrast, in sample 26#, ZnO is interspersed among the TiO_2_ nanoparticles in rod-like forms of varying lengths, as shown in Figure 7c. The excellent dispersibility creates a relatively loose overall structure, increasing the exposure of active sites and thereby enhancing the photocatalytic performance of the composite. Additionally, Figure 7d reveals that numerous TiO_2_ particles adhere to the ZnO rod surfaces, inhibiting stacking and agglomeration of TiO_2_ nanoparticles and providing more active sites for photocatalytic reactions [29]. Furthermore, the EDS spectrum of the selected region in Figure 7d confirms that the sample consists of O, Ti, and Zn elements, verifying the existence of a TiO_2_-ZnO heterostructure. The S element detected in the spectrum originates from the Zn source material, ZnSO_4_.

N_2_ adsorption–desorption tests were conducted on TiO_2_ and sample 26#. The BET surface areas were found to be 8.52 m^2^/g and 8.31 m^2^/g, respectively, indicating that the pore structures of both materials are at a similar level and have a minor impact on catalytic activity [30]. The slightly lower BET surface area of sample 26# compared to TiO_2_ may be attributed to the incorporation of Zn, which forms long rod-like particles that combine with the TiO_2_ particles.

**Figure 8:**
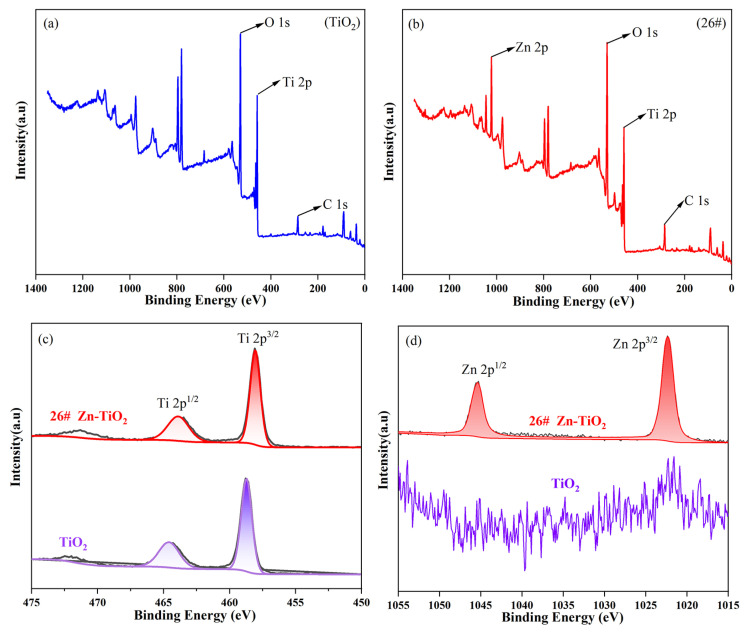
XPS spectra of pure TiO_2_ and sample 26#: (**a**,**b**) full spectrum; (**c**) Ti 2p; (**d**) Zn 2p.

XPS was employed to characterize sample 26# and TiO_2_, and the results are illustrated in Figure 8. Compared with Figure 8a, a distinct Zn 2p peak appears at 1022 eV in Figure 8b, confirming the presence of Zn in sample 26#. Figure 8c,d show the comparative XPS spectra of Ti 2p and Zn 2p for sample 26# and TiO_2_, respectively.

As shown in Figure 8c, the Ti 2p peaks of sample 26# exhibit a slight rightward shift (a decrease in binding energy). This can be attributed to the electronic interaction between the introduced Zn and Ti, which increases the electron density around Ti atoms, thereby reducing the energy required for photoelectrons to escape from the sample [17]. In Figure 8d, two peaks are observed at 1022 eV and 1045 eV, corresponding to the Zn 2p^3/2^ and Zn 2p^1/2^ orbitals, respectively. This indicates that zinc exists in the form of Zn^2+^ within the composite material. Conversely, as pure TiO_2_ contains no Zn element, no Zn 2p peaks are observed in its spectrum.

### 3.4. Photocatalytic Performance of the Optimal Sample

**Figure 9:**
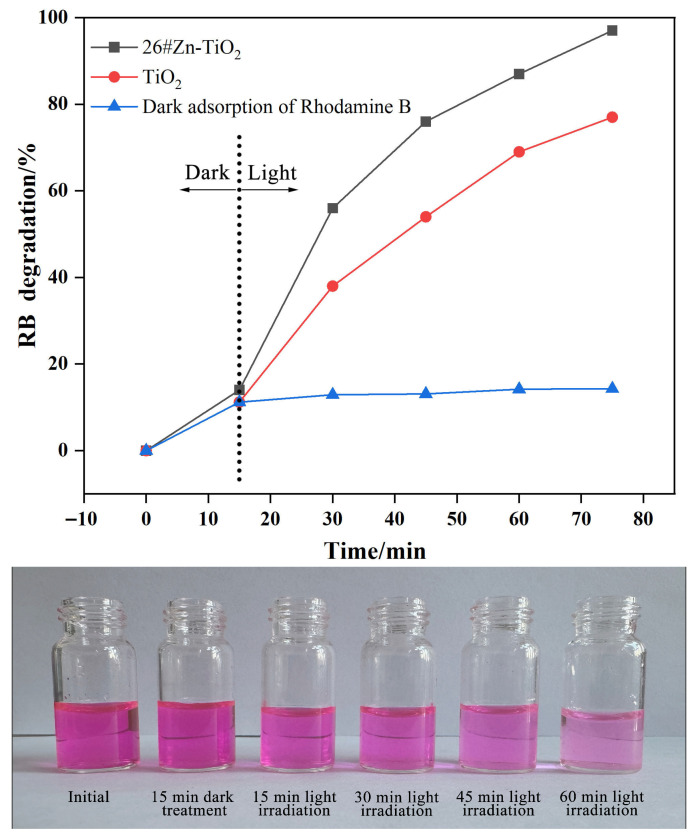
Photocatalytic degradation curves of RB with TiO_2_ and 26# Zn-TiO_2_ under UV irradiation, and photographs of RB solutions after different times of catalytic reaction with 26# Zn-TiO_2_.

The photocatalytic performance of sample 26# for the degradation of RB under UV irradiation was evaluated using a UV–Vis spectrophotometer, with TiO_2_ serving as the control group. The variation in the RB degradation over time is shown in Figure 9. After 15 min of dark treatment, the RB degradation rates of both samples were relatively low, indicating that the dye removal effect achieved by stirring alone is limited [31]. Upon exposure to 365 nm UV light, the degradation rate of the RB solution rapidly increased with the increase in irradiation time. Throughout the entire reaction process, the degradation rate of sample 26# was consistently higher than that of TiO_2_. After 60 min of irradiation, the degradation efficiency of the RB solution by sample 26# reached 97%, demonstrating superior photocatalytic activity compared to TiO_2_ and all other Zn-TiO_2_ samples from the orthogonal experiments.

**Figure 10:**
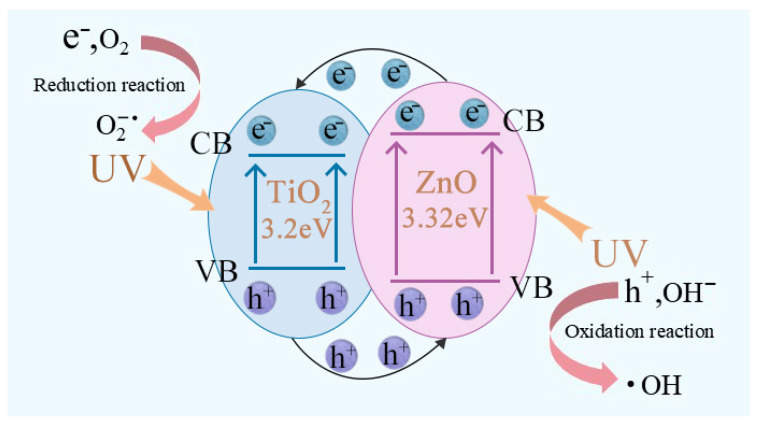
Schematic illustration of the heterojunction structure and charge carrier transfer of the Zn-TiO_2_ catalyst.

Based on the analysis and discussion of the characterization results presented above, the enhancement in catalytic performance is primarily attributed to the formation of a heterostructure between TiO_2_ and ZnO derived from Zn-doping. When irradiated with UV light, electrons in the valence band (VB) of TiO_2_ are excited to its conduction band (CB), leaving holes (h^+^) in the VB; similarly, electrons in the VB of ZnO are excited to its CB, generating electron–hole pairs. Due to the fact that the CB of ZnO has a greater negative potential than that of TiO_2_, the photogenerated electrons in the CB of ZnO spontaneously transfer to the CB of TiO_2_, where they undergo a reduction reaction with adsorbed O_2_ on the catalyst surface. Meanwhile, the VB of ZnO has a greater positive potential than that of TiO_2_, and the holes in TiO_2_ migrate to the VB of ZnO and react with adsorbed OH^–^ via an oxidation process [29,32]. The detailed mechanism is illustrated in Figure 10. This bidirectional migration of electrons and holes across the heterostructure, coupled with the spatial separation of redox reactions, effectively inhibits the recombination of photogenerated electron–hole pairs. As a result, the utilization efficiency of charge carriers is significantly improved, thereby greatly enhancing the catalytic degradation performance of the photocatalyst for RB [33]. Furthermore, the introduced rod-like ZnO particles optimize the microstructure of the TiO_2_ catalyst, providing more active sites for the catalytic reaction and making a positive contribution to the improvement of catalytic activity.

### 3.5. Catalytic Performance of Cement Mortar-Based Photocatalyst Composites

**Figure 11:**
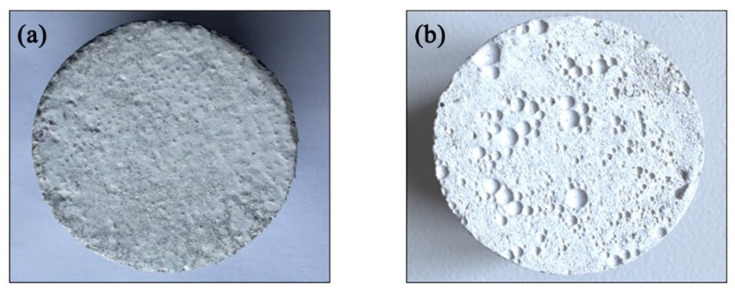
Photographs of (**a**) CM-based and (**b**) ACM-based photocatalyst composite.

The TiO_2_ and 26# Zn-TiO_2_ catalysts were immobilized onto the surface of cement mortar (CM) using the slurry coating method to obtain TiO_2_/CM and Zn-TiO_2_/CM composites. Similarly, the TiO_2_ and 26# Zn-TiO_2_ catalyst were loaded onto the surface of air-entrained composite mortar (ACM), yielding TiO_2_/ACM and Zn-TiO_2_/ACM composites, as shown in Figure 11. It can be observed that, compared to the relatively dense surface of the CM-based composites, the surface of the ACM-based composites exhibits significantly higher porosity. This indicates that the incorporation of an air-entraining agent increases the porosity and significantly improves the pore structure of the surface layer of cement mortar, thereby optimizing the dispersibility and adhesion of the photocatalysts on the substrate surface.

**Figure 12:**
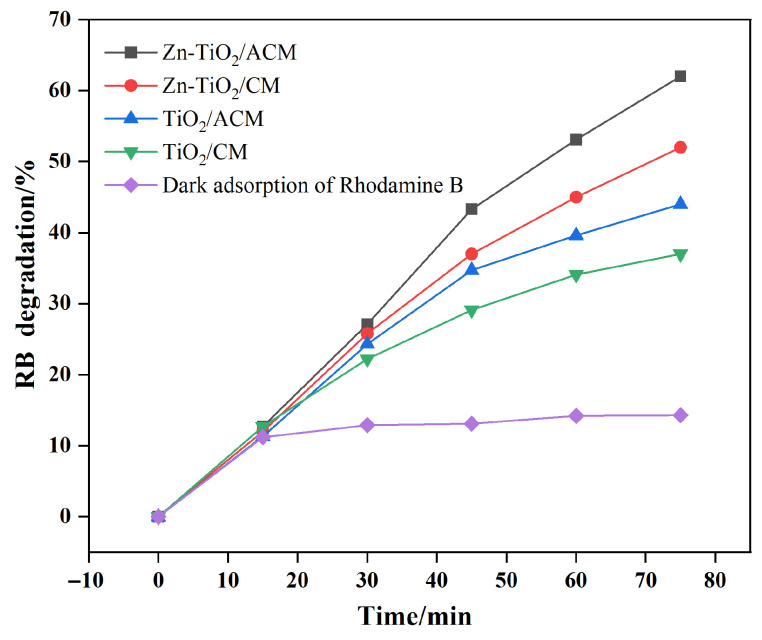
Photocatalytic degradation curves of RB for the four types of cement mortar-based photocatalyst composites under UV irradiation.

The photocatalytic performance of the four aforementioned composite materials on RB solution was investigated under UV irradiation. The variation in the RB degradation over time is shown in Figure 12. After 60 min of irradiation, the degradation rate of RB with TiO_2_/CM was 37%, whereas the degradation rate with Zn-TiO_2_/CM reached 52%, representing an increase of 41%. This result indicates that after immobilization on the CM substrate surface, the photocatalytic activity of Zn-TiO_2_ remains significantly higher than that of TiO_2_. However, compared to the powder catalysts, the overall activity decreased markedly when coated onto building material surfaces. This is because the active sites located at the binding interface and within the coating layer are unable to come into contact with the pollutants, preventing them from participating in the catalytic process. In contrast, the degradation rates of TiO_2_/ACM and Zn-TiO_2_/ACM reached 44% and 62%, respectively, after 60 min of irradiation. These values represent improvements of 13.5% and 19.2% compared to TiO_2_/CM and Zn-TiO_2_/CM. It is evident that the ACM substrate facilitates the effective realization of the photocatalyst’s performance. The optimization of the substrate structure improves its interfacial bonding with the catalyst, preventing the masking of active sites caused by catalyst agglomeration and ensuring sufficient contact with pollutants.

**Figure 13:**
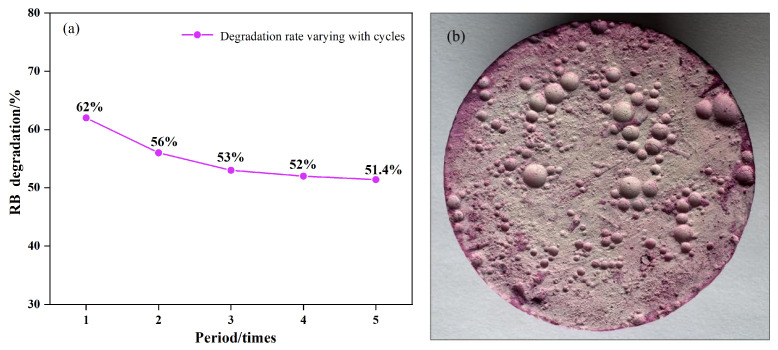
Cyclic catalytic degradation of RB by the Zn-TiO_2_/ACM composite under UV irradiation: (**a**) degradation rate curves and (**b**) surface photograph after reaction.

To evaluate the retention of the photocatalytic performance of the Zn-TiO_2_/ACM composite during long-term use, cyclic tests were employed to investigate its stability. By repeating multiple photocatalytic degradation reactions, phenomena such as catalyst deactivation, leaching, and structural damage of the composite can be intuitively reflected. The experimental procedure was consistent with the catalytic performance test for cement mortar-based photocatalyst composites. After each reaction cycle, the Zn-TiO_2_/ACM composite was removed and rinsed with deionized water to remove residual pollutants. Subsequently, it was dried at 60 °C for 2 h before the next catalytic reaction. This process was repeated for five cycles, and the degradation rates of RB after 60 min of UV irradiation were measured for each cycle, as shown in Figure 13a. The Zn-TiO_2_/ACM composite exhibited good stability over five consecutive cycles. The initial degradation rate was 62%. As the number of cycles increased, the degradation rate decreased slowly, and the rate of decline gradually diminished, eventually reaching 51.4% in the final cycle. After five cycles of cyclic aging experiments, the photocatalytic performance still remained at 83% of the initial value. The drop in the degradation rate during the second cycle might be due to the flushing of the first solution, which caused the Zn-TiO_2_ particles that were weakly bonded to the functionalized surface layer to fall off, thereby reducing the active sites for the photocatalytic reaction. With the increase in cycles, the number of active sites tended to stabilize. However, the coverage effect of residual pollutants hindered the contact between the photogenerated charge carriers on the surface photocatalyst and the target pollutants, leading to a gradual decline in the degradation rate. Nevertheless, no abrupt drop in the degradation rate was observed throughout the process, indicating that the Zn-TiO_2_/ACM composite possesses excellent stability. The surface morphology of the Zn-TiO_2_/ACM composite after the cyclic tests is shown in Figure 13b. Compared with Figure 11b, the overall surface color changed from white to light purplish-red, and slight deposition or coverage was visible in localized areas. This is due to the adsorption of residual dye into the surface pores of the material during the cyclic experiments. Importantly, the pore structure remained clear, and no large-scale collapse or spalling was observed.

## 4. Conclusions

In this study, Zn-TiO_2_ photocatalysts were successfully synthesized via a hydrothermal method. The optimal preparation conditions were determined through orthogonal experiments as follows: hydrothermal temperature of 120 °C, reaction time of 10 h, and Ti/Zn molar ratio of 100:5. Under these conditions, the obtained Zn-TiO_2_ photocatalyst exhibited good particle dispersion and formed a stable heterojunction structure, which could provide more active sites for the photocatalytic reaction and effectively inhibited the recombination of photogenerated charge carriers. Its photocatalytic degradation efficiency for RB was 26% higher than that of TiO_2_ under identical conditions.

By loading the Zn-TiO_2_ photocatalyst onto cement mortar substrates of different types, novel green building materials capable of degrading pollutants—Zn-TiO_2_/CM and Zn-TiO_2_/ACM—were successfully developed. The surface of the CM substrate was relatively smooth and dense, which was unfavorable for catalyst loading and adhesion. As a result, the degradation efficiency of the Zn-TiO_2_/CM composite was approximately half that of the powder catalyst. In contrast, the ACM substrate featured a double-layer structure, and its surface pore structure was optimized by the addition of an air-entraining agent. Immobilizing the catalyst on the ACM surface significantly enhanced the photocatalytic performance of the building material, with the degradation efficiency of the Zn-TiO_2_/ACM composite being 19.2% higher than that of Zn-TiO_2_/CM. The photocatalytic performance still remained at 83% of the initial value after five cycles of cyclic aging experiments. This demonstrated that the Zn-TiO_2_/ACM composite exhibited excellent catalytic stability and reusability and had broad application prospects in the field of green building materials and environmental remediation.

This study only investigated the photocatalytic degradation performance of Zn-TiO_2_ composite materials based on air-entrained composite mortar under UV conditions for RB. Further research is needed to enhance the intrinsic performance of the Zn-TiO_2_ catalyst under visible light conditions and to test the long-term degradation ability of the composite material for dye mixtures that are closer to actual use conditions. In addition, future investigations should focus on the influence of the types and dosages of air-entraining agents on the surface structure of the cement mortar, in order to improve the comprehensive performance of photocatalytic building materials.

## Figures and Tables

**Table 1:** Factors and levels of the orthogonal experiment.

Level	Temperature (°C)	Time (h)	Ti:Zn (mol)
1	100	4	100:5
2	120	6	100:10
3	140	8	100:15
4	160	10	100:20
5	180	12	100:25

**Table 2:** L_25_(5^3^) detailed design of orthogonal experiment.

Factor	Temperature (°C)	Time (h)	Ti:Zn (mol)
1#	100	4	100:5
2#	100	6	100:10
3#	100	8	100:15
4#	100	10	100:20
5#	100	12	100:25
6#	120	4	100:10
7#	120	6	100:15
8#	120	8	100:20
9#	120	10	100:25
10#	120	12	100:5
11#	140	4	100:15
12#	140	6	100:20
13#	140	8	100:25
14#	140	10	100:5
15#	140	12	100:10
16#	160	4	100:20
17#	160	6	100:25
18#	160	8	100:5
19#	160	10	100:10
20#	160	12	100:15
21#	180	4	100:25
22#	180	6	100:5
23#	180	8	100:10
24#	180	10	100:15
25#	180	12	100:20

**Table 3:** Range analysis results.

Item	Level	Factor 1 (Hydrothermal Temperature)	Factor 2 (Reaction Time)	Factor 3 (Ti/Zn Molar Ratio)
K value	1	296.00	389.00	443.00
	2	453.00	377.00	357.00
	3	373.00	379.00	390.00
	4	399.00	395.00	354.00
	5	358.00	339.00	335.00
Kavg value	1	59.20	77.80	88.60
	2	90.60	75.40	71.40
	3	74.60	75.80	78.00
	4	79.80	79.00	70.80
	5	71.60	67.80	67.00
Optimal level		2	4	1
Range R		31.40	11.20	21.60

## Data Availability

The original contributions presented in this study are included in the article. Further inquiries can be directed to the corresponding authors.
